# How and why does tomato accumulate a large amount of GABA in the fruit?

**DOI:** 10.3389/fpls.2015.00612

**Published:** 2015-08-10

**Authors:** Mariko Takayama, Hiroshi Ezura

**Affiliations:** The Ezura Laboratory, Graduate School of Life and Environmental Sciences, University of Tsukuba, Tsukuba, Japan

**Keywords:** GABA, GABA shunt, tomato, fruit, metabolism

## Abstract

Gamma-aminobutyric acid (GABA) has received much attention as a health-promoting functional compound, and several GABA-enriched foods have been commercialized. In higher plants, GABA is primarily metabolized via a short pathway called the GABA shunt. The GABA shunt bypasses two steps (the oxidation of α-ketoglutarate to succinate) of the tricarboxylic acid (TCA) cycle via reactions catalyzed by three enzymes: glutamate decarboxylase, GABA transaminase, and succinic semialdehyde dehydrogenase. The GABA shunt plays a major role in primary carbon and nitrogen metabolism and is an integral part of the TCA cycle under stress and non-stress conditions. Tomato is one of the major crops that accumulate a relatively high level of GABA in its fruits. The GABA levels in tomato fruits dramatically change during fruit development; the GABA levels increase from flowering to the mature green stage and then rapidly decrease during the ripening stage. Although GABA constitutes up to 50% of the free amino acids at the mature green stage, the molecular mechanism of GABA accumulation and the physiological function of GABA during tomato fruit development remain unclear. In this review, we summarize recent studies of GABA accumulation in tomato fruits and discuss the potential biological roles of GABA in tomato fruit development.

## Introduction

**FIGURE 1:**
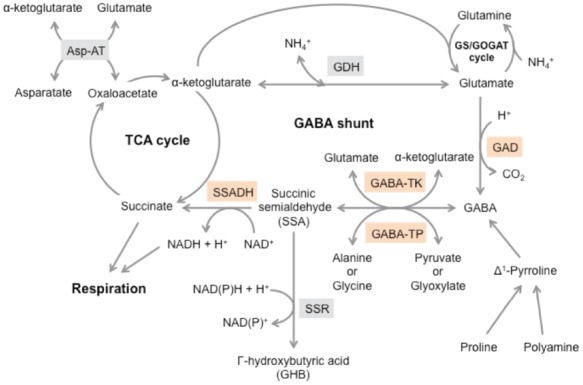
**GABA metabolism and related pathways.** GAD, glutamate decarboxylase; GABA-TK, α-ketoglutarate-dependent GABA transaminase; GABA-TP, pyruvate-dependent GABA transaminase; SSA, succinic semialdehyde; SSADH, succinic semialdehyde dehydrogenase; GDH, glutamate dehydrogenase; SSR, succinic semialdehyde reductase; Asp-AT, aspartate aminotransferase.

Gamma-aminobutyric acid (GABA), a four-carbon non-proteinogenic amino acid, is widely found in animals, plants and bacteria. In humans, GABA functions as an inhibitory neurotransmitter in the central nervous system (Owens and Kriegstein, 2002). It has also been reported that GABA is effective at reducing blood pressure, inducing relaxation and enhancing immunity when administered orally (Inoue et al., 2003; Abdou et al., 2006). Thus, GABA has received much attention as a health-promoting functional compound, and several GABA-enriched foods have been commercialized. In higher plants, GABA is primarily metabolized via a short pathway called the GABA shunt, which bypasses two steps (the oxidation of α-ketoglutarate to succinate) of the tricarboxylic acid (TCA) cycle (Satya-Narayan and Nair, 1990; Bouché and Fromm, 2004; Figure 1). In this pathway, GABA is synthesized from glutamate in a reaction catalyzed by the enzyme glutamate decarboxylase (GAD) and subsequently catabolized to succinate through two consecutive reactions catalyzed by GABA transaminase (GABA-T) and succinic semialdehyde dehydrogenase (SSADH). Previous studies have suggested that the GABA shunt is involved in multiple physiological responses, such as the regulation of cytosolic pH, maintenance of carbon/nitrogen balance, defense against insects, protection from oxidative stress, and production of energy (Bouché and Fromm, 2004; Fait et al., 2008). Moreover, the GABA level rapidly increases in plant tissues subjected to diverse stimuli, including heat shock, mechanical stimulation, hypoxia, and phytohormones (Shelp et al., 1999).

Tomato (*Solanum lycopersicum*) is a major crop produced worldwide. Tomato fruits are a significant food resource and have been considered an experimental model for studying the physiology, development and ripening of fleshy fruits (Steinhauser et al., 2010; Osorio et al., 2011). Tomatoes accumulate a relatively high level of GABA in the fruits (Matsumoto et al., 1997). In several cultivated tomatoes, drastic changes in GABA levels have been observed during fruit development; the GABA level increases to the mature green stage and subsequently rapidly decreases during the ripening stage (Rolin et al., 2000; Carrari et al., 2006; Akihiro et al., 2008; Saito et al., 2008; Osorio et al., 2011). In cherry tomatoes, GABA is reported to constitute up to 50% of the free amino acids at the mature green stage (Rolin et al., 2000). Despite the large accumulation, the molecular mechanism of GABA accumulation and the physiological function of this amino acid during tomato fruit development remain elusive. Elucidating these topics would help us to gain a better understanding of plant physiology, particularly in fruits. In this review, we summarize recent studies concerning GABA accumulation in tomato fruits and discuss the potential biological roles of GABA in tomato fruit development.

## GABA Biosynthesis

In plants, GABA is primarily synthesized via the cytosolic enzyme GAD, which catalyzes the irreversible conversion of glutamate to GABA and CO_2_ (Figure 1). A plant GAD gene was first isolated from *Petunia hybrida* (Baum et al., 1993), and subsequently, several GAD homologues have been identified in various plant species (Ling et al., 1994; Snedden et al., 1995; Turano and Fang, 1998; Yevtushenko et al., 2003). Unlike its counterparts in animals and bacteria, most plant GADs possess a calcium/calmodulin (Ca^2+^/CaM) binding domain (CaMBD) at the C-terminus. *In vitro* studies have shown that GAD activity is stimulated through a low pH or the binding of Ca^2+^/CaM to the CaMBD at physiological pH (Snedden et al., 1996; Gut et al., 2009). In addition, transgenic studies revealed that the removal of the CaMBD increased GABA accumulation in plants (Baum et al., 1996; Akama and Takaiwa, 2007). Thus, it is considered that the CaMBD acts as a negative regulator/autoinhibitory domain in the absence of Ca^2+^/CaM, and the negative regulation is relieved through the binding of Ca^2+^/CaM.

In tomato, GAD gene was first cloned in 1995. Gallego et al. (1995) isolated *ERT D1*, a gene encoding a putative GAD protein, from a cDNA library of the pericarp of cv. “Ailsa Craig.” Similar to other plant GADs, ERT D1 protein contained a putative CaMBD. It was also revealed that *ERT D1* mRNA levels peaked at the beginning of fruit ripening (Gallego et al., 1995). Subsequently, Kisaka et al. (2006) isolated *GAD-19*, a gene encoding another GAD protein, from tomato roots. The antisense suppression of this gene in tomato plants resulted in the production of fruits with decreased levels of GAD mRNA. However, the GABA level in these fruits was not significantly decreased compared with the WT levels, although increased levels of total free amino acids (particularly glutamate, which is the precursor to GABA) were observed (Kisaka et al., 2006). Subsequently, Akihiro et al. (2008) isolated three GAD genes, designated *SlGAD1*, *SlGAD2*, and *SlGAD3*, from the immature fruits of cv. “Micro-Tom.” Because the amino acid sequences of *SlGAD1* and *ERT D1* are precisely identical, *SlGAD1* is considered an allele of *ERT D1* (Akihiro et al., 2008). However, neither *SlGAD2* nor *SlGAD3* share precisely identical sequences with *GAD-19*, although blast database searches indicate that *SlGAD2* has the highest homology to *GAD-19* (95% identity and 98% similarity in amino acid sequences). Among the three *SlGAD*s isolated from cv. “Micro-Tom,” *SlGAD2* and *SlGAD3* appear to play a major role in GABA production in tomato fruits, as the expression levels of *SlGAD2* and *SGAD3* are positively correlated with the GABA accumulation during fruit development (Akihiro et al., 2008). Additionally, transgenic tomato plants, in which *SlGAD2* or *SlGAD3* was specifically suppressed, accumulated a significantly decreased level of GABA in the fruits, whereas *SlGAD1*-suppressed plants produced fruits with normal levels of GABA (Takayama et al., 2015). Moreover, in triple *SlGADs*-suppressed plants, the fruit GABA level decreased to less than 10% of the WT level (Takayama et al., 2015), suggesting that the main route of GABA biosynthesis in tomato fruits is the decarboxylation of glutamate via GAD enzymes under normal growth conditions.

Enhanced GABA accumulation in tomato fruits has been observed in plants grown under salinity conditions or in fruits stored under 10% CO_2_ or under low O_2_ conditions after harvesting (Deewatthanawong et al., 2010; Yin et al., 2010; Mae et al., 2012). Although the expression levels of *SlGAD2* and *SlGAD3* were not enhanced in the fruits under salinity conditions (Yin et al., 2010), those in fruits stored under 10% CO_2_ or low O_2_ conditions were up-regulated (Deewatthanawong et al., 2010; Mae et al., 2012). These results suggest that *SlGAD2* and *SlGAD3* are responsive to some types of stresses. It has been suggested that stress-induced GABA accumulation in plant cells reflects increases in cytosolic H^+^, Ca^2+^ or glutamate levels, as these factors stimulate GAD activity (Shelp et al., 1999). However, in tomato, stress-induced GAD activity might also be regulated at the transcriptional level. Although it is reported that GABA can also be formed from polyamines or proline via a Δ^1^-pyrroline intermediate formation in response to abiotic stresses (Flores and Filner, 1985; Shelp et al., 2012; Yang et al., 2013; Signorelli et al., 2015), the contribution of these pathways in tomato fruits is still unclear.

## GABA Catabolism

In many organisms, GABA is first converted to SSA via a transamination reaction through GABA-T (Figure 1). According to substrate specificity, the GABA-T enzyme can be divided into two types: α-ketoglutarate-dependent GABA-T (GABA-TK) and pyruvate-dependent GABA-T (GABA-TP). The former uses α-ketoglutarate as an amino group acceptor to generate glutamate, whereas the latter uses pyruvate to generate alanine (Bouché and Fromm, 2004). It is clear that GABA-TP also has glyoxylate-dependent GABA-T (GABA-TG) activity, which uses glyoxylate as an amino group acceptor to generate glycine (Clark et al., 2009a,b; Shimajiri et al., 2013; Trobacher et al., 2013). GABA-TK is exclusively utilized in bacteria, yeast, fungi and mammals (Satya-Narayan and Nair, 1990). However, both GABA-TK and GABA-TP activities have been detected in plant crude extracts (Shelp et al., 1995; Van Cauwenberghe and Shelp, 1999; Bartyzel et al., 2003), although only the GABA-TP gene has been isolated from plants (Van Cauwenberghe et al., 2002). Tomato is one of the species exhibiting both GABA-TK and GABA-TP activities. Although most previously investigated plants have shown lower GABA-TK activity than GABA-TP activity, Akihiro et al. (2008) detected a significantly higher level of GABA-TK activity in tomato fruits after the breaker stage. Comparison analyses between ordinary and GABA rich cultivars revealed a negative correlation between GABA contents and GABA-TK activity during fruit development (Akihiro et al., 2008). Similar trends were also observed in the tomato fruits stored under low O_2_ conditions, in which GABA levels were increased compared with those in fruits stored under control (air) conditions (Mae et al., 2012). These observations suggest that GABA-TK plays a major role in catabolism in tomato fruits. However, Clark et al. (2009b) presented a different view, as no GABA-TK activity was detected in assays using the cell-free extracts from the fruits of cv. “Micro-Tom,” which is the same cultivar used in Akihiro et al. (2008). Moreover, Clark et al. (2009b) observed higher levels of GABA-TP activity in tomato fruits. Thus, these authors noted the possibility that the previous study detected artificial GABA-TK activity and concluded that pyruvate/glyoxylate-dependent GABA-T activity probably accounts for the GABA catabolism observed in tomato fruits. Currently, three GABA-T genes, designated *SlGABA-T1*, *SlGABA-T2*, and *SlGABA-T3*, have been isolated from tomato cv. “Micro-Tom” (Akihiro et al., 2008; Clark et al., 2009b). Although the encoded proteins are localized to distinct subcellular compartments [i.e., mitochondrion (SlGABA-T1), cytosol (SlGABA-T2), or plastid (SlGABA-T3)], all three isoforms are characterized as GABA-TPs, which exhibit pyruvate/glyoxylate-dependent GABA-T activity (Clark et al., 2009b). To clarify the physiological function of these *SlGABA-T* isoforms in tomato fruits, Koike et al. (2013) conducted loss-of-function analyses using RNA interference (RNAi) transgenic lines with suppressed *SlGABA-T* genes. In this study, increased GABA accumulation was observed in the fruits of *SlGABA-T1*-suppressed lines (1.3–2.0 times higher in mature green fruits and 6.8–9.2 times higher in red fruits), whereas almost no correlation was observed between the GABA content and the expressions of *SlGABA-T2* and *SlGABA-T3* (Koike et al., 2013). Considering that the enzymatic activity of SlGABA-T1 is highest among the three isoforms in tomato fruits (Clark et al., 2009b), Koike et al. (2013) concluded that pyruvate- and glyoxylate-dependent SlGABA-T1 is the essential isoform for GABA reduction in the ripening fruits.

In plants, GABA-derived SSA is catabolized via the NAD^+^-dependent enzyme SSADH, which oxidizes SSA to succinate concomitantly with NADH production in mitochondria (Breitkreuz and Shelp, 1995; Busch and Fromm, 1999; Figure 1). Alternatively, SSA can also be catabolized to γ-hydroxybutyric acid (GHB) through enzymes with SSA reductase (SSR) activity (Breitkreuz et al., 2003; Hoover et al., 2007; Simpson et al., 2008; Figure 1). The former pathway provides substrates (succinate and NADH) for the mitochondrial respiratory machinery, which produces ATP as a final product (Bouché and Fromm, 2004). It is also known that SSADH activity is highly sensitive to the energy status in mitochondria (Busch and Fromm, 1999). Thus, under stress conditions in which the NAD^+^:NADH ratio is low, SSADH activity would be inhibited, resulting in the accumulation of SSA and feedback inhibition of GABA-T (Busch and Fromm, 1999; Van Cauwenberghe and Shelp, 1999). However, the pathway from SSA to GHB is stimulated under stress conditions and likely functions in stress tolerance through the detoxification of SSA (Breitkreuz et al., 2003; Allan et al., 2008). In tomato, one SSADH gene (*SlSSADH*) and two SSR genes (*SlSSR1*, *SlSSR2*) have been isolated (Akihiro et al., 2008). *SlSSADH* is expressed in fruits at all developmental stages, and the expression of this gene is poorly correlated with the GABA contents (Akihiro et al., 2008). However, the expression of *SlSSR1* gene is slightly higher in red fruits than in breaker fruits, whereas *SlSSR2* expression is higher in breaker fruits compared with red fruits (Deewatthanawong et al., 2010). However, the biochemical properties of the encoded proteins and their contribution to GABA accumulation in tomato fruits remain unclear.

## The Potential Role of GABA Metabolism in Tomato Plants

In plants, GABA metabolism is involved in a wide range of physiological processes. For example, *pop2*, an *Arabidopsis* GABA-T-deficient mutant, is defective in the guidance and growth of pollen tubes (Palanivelu et al., 2003; Renault et al., 2011). *Arabidopsis* SSADH-deficient mutants exhibit severe dwarfism and necrotic lesions under the standard light conditions (Bouché et al., 2003). Additionally, these mutants exhibit the enhanced accumulation of reactive oxygen intermediates and cell death under environmental stresses (Bouché et al., 2003). Another *Arabidopsis* SSADH-deficient mutant, *enf1*, forms both abaxialized and adaxialized leaves (Toyokura et al., 2011). Notably, the abnormal phenotypes observed in the two different studies of *ssadh* mutants (Bouché et al., 2003; Toyokura et al., 2011) are both suppressed through an additional mutation in GABA-T, suggesting that these phenotypes reflect the accumulation of SSA or close derivatives, such as GHB (Ludewig et al., 2008; Toyokura et al., 2011). In tomatoes, several abnormalities have also been observed when GABA metabolism is altered. For example, *SlGABA-T1*-suppressed plants exhibited severe infertility, and both *SlGABA-T1*- and *SlGABA-T3*-suppressed plants exhibited dwarf phenotypes (Koike et al., 2013). Moreover, *SlSSADH*-suppressed plants show a dwarf phenotype, curled leaves and enhanced ROS accumulation under normal conditions (Bao et al., 2014). Interestingly, when tomato seedlings were grown under salt stress (200 mM NaCl), *SlSSADH*-suppressed plants exhibited significantly higher shoot biomass levels and increased chlorophyll contents and photosynthetic rates compared with control plants (Bao et al., 2014). However, *SlGADs*-suppressed plants and *SlGABA-Ts*-suppressed plants are more sensitive to salt stress, resulting in reduced biomass and the total collapse of tissue (Bao et al., 2014). These observations indicate that GABA shunt is involved in salt stress tolerance in tomato plants. Moreover, GABA shunt has been implicated in resistance against *Botrytis cinerea*, as GABA shunt genes are up-regulated in the leaves of the *B. cinerea*-resistant mutant, *sitiens*, and the exogenous application of GABA decreases susceptibility to *B. cinerea* in wild-type leaves (Seifi et al., 2013).

**FIGURE 2:**
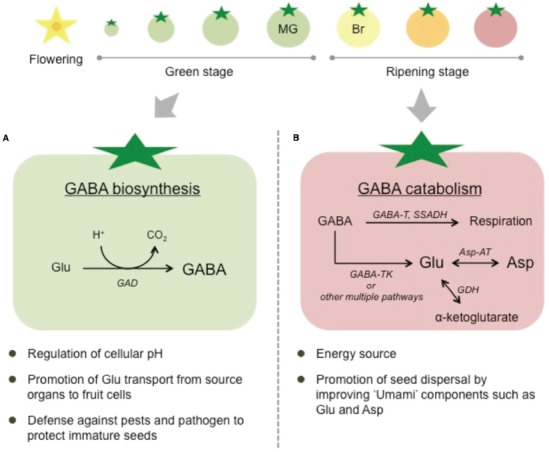
**Potential roles of GABA in tomato fruits. (A)** Fruits at the early developmental (green) stage when GABA is biosynthesized. **(B)** Fruits at the ripening stage when GABA is catabolized. MG, mature green; Br, breaker; GAD, glutamate decarboxylase; GABA-T (K), (α-ketoglutarate-dependent) GABA transaminase; SSADH, succinic semialdehyde dehydrogenase; Asp-AT, aspartate aminotransferase; Glu, glutamate; Asp, aspartate.

As described above, effects of impaired GABA metabolism on tomato plants have been increasingly reported. However, little is known about the function of GABA and the metabolism of this amino acid in fruits. Previous studies have suggested that GABA production during fruit development might contribute to the regulation of cellular pH (Rolin et al., 2000). During tomato fruit development, organic acids are continuously synthesized from unloaded sucrose, coupled with proton production. Overaccumulation of protons would cause an intracellular acidification, but the intracellular pH is probably regulated by ATP-driven proton pumps that extrude intracellular protons out of the cytoplasm, or by the proton-consuming decarboxylation of organic acids. Because GAD reaction requires protons, it might act as a sink for excess protons, preventing intracellular acidification (Rolin et al., 2000; Figure 2A). Moreover, the GAD reaction also promotes glutamate transport. In cherry tomatoes, glutamate is translocated through phloem sap and unloaded in fruits. The unloaded glutamate is subsequently transported symplastically or taken up through a proton symport mechanism across the membrane. In the latter transport mechanism, glutamate and protons are cotransported into the cytosol, thereby promoting cytoplasmic acidosis and the depolarization of the plasma membrane. Thus, continuous GABA accumulation during fruit development reflects the continuous GAD reaction, which potentially maintains glutamate transport through the consumption of excess protons (Snedden et al., 1992; Rolin et al., 2000). In addition, accumulated GABA in tomato fruits functions as an energy source, as ^14^C-labeled CO_2_ was discharged from fruits fed ^14^C-labeled GABA, indicating that GABA is utilized as a substrate for respiration (Yin et al., 2010; Figure 2B). Indeed, GABA shunt also functions as an alternative pathway for the production of succinate (the substrate for respiration) in tomato leaves when the enzyme of the TCA cycle is impaired (Studart-Guimarães et al., 2007). However, recent findings suggest that GABA metabolism has little effect on tomato fruit development under normal conditions, as the fruits of RNAi transgenic plants targeting the three *SlGADs* exhibited normal development, although the enzymatic activity of GAD and the GABA content in fruits were dramatically decreased (Takayama et al., 2015). Similarly, RNAi transgenic plants targeting *SlGABA-T* also produced normal fruits, although the GABA levels in red fruits were 6.8–9.2 times higher than those in wild-type controls (Koike et al., 2013). Therefore, GABA metabolism in tomato fruits might be involved in stress tolerance, similar to other plants. Another possibility is that GABA contributes to tomato seed dispersal through changes in the amino acid composition during fruit development. Because GABA functions in defense against pests and pathogens (Bown et al., 2006; Seifi et al., 2013), GABA accumulation in fruits at the early developmental stage might protect immature seeds (Figure 2A). However, the GABA levels in fruits rapidly decline during the ripening stage, when seeds have already matured. In parallel, the levels of glutamate and/or aspartate, which provide the “Umami taste,” dramatically increase during the ripening stage. These changes in the amino acid composition might attract insects and animals, resulting in successful seed dispersal (Figure 2B). The increases in glutamate and/or aspartate during fruit ripening have been well characterized in various cultivars (Rolin et al., 2000; Akihiro et al., 2008; Koike et al., 2013). The increase in glutamate probably reflects the increase in glutamate dehydrogenase (GDH) and GABA-TK activities during the ripening stage and the decreased consumption of glutamate through GAD, which is almost undetectable in ripe fruits (Sorrequieta et al., 2010; Ferraro et al., 2015; Figure 1). On the other hand, aspartate is synthesized from glutamate through aspartate aminotransferase (Figure 1). In GABA-rich cultivars, lower levels of glutamate and aspartate have been observed in ripening fruits (Akihiro et al., 2008), suggesting that GABA catabolism contributes to the accumulation of glutamate and glutamate-derived aspartate in ripening fruits. Furthermore, Snowden et al. (2015) recently identified a tonoplast-localized glutamate/aspartate/GABA exchanger (SlCAT9) in tomato fruits. As overexpression of the *SlCAT9* gene strongly influences the accumulation of glutamate, aspartate, and GABA during tomato fruit development, it is suggested that the intracellular transport of amino acids between vacuole and cytosol is also a major determinant of their accumulation in ripening fruits (Snowden et al., 2015). Although the pathway involving the conversion from GABA to glutamate remains uncertain, GABA catabolism might play a crucial role in the determination of tomato fruit taste during ripening.

### Conflict of Interest Statement

The authors declare that the research was conducted in the absence of any commercial or financial relationships that could be construed as a potential conflict of interest.
